# Effectiveness of a Virtual-Reality-Based Self-Help Intervention for Lowering the Psychological Burden during the COVID-19 Pandemic: Results from a Randomized Controlled Trial in Iran

**DOI:** 10.3390/jcm12052006

**Published:** 2023-03-02

**Authors:** Sharareh Farahimanesh, Silvia Serino, Cosimo Tuena, Daniele Di Lernia, Brenda K. Wiederhold, Luca Bernardelli, Giuseppe Riva, Alireza Moradi

**Affiliations:** 1 Institute for Cognitive and Brain Sciences, Shahid Beheshti University, Tehran 1983969411, Iran; 2 Institute for Cognitive Science Studies, Tehran 1658344575, Iran; 3 Department of Psychology, Università Cattolica del Sacro Cuore, 20123 Milan, Italy; 4 Applied Technology for Neuropsychology Lab, IRCCS Istituto Auxologico Italiano, 20145 Milan, Italy; 5 Humane Technology Lab, Università Cattolica del Sacro Cuore, 20123 Milan, Italy; 6 Virtual Reality Medical Center, La Jolla, CA 92037, USA; 7 Virtual Reality Medical Institute, 1200 Brussels, Belgium; 8 Become-Hub, 20100 Milan, Italy; 9 Faculty of Psychology & Education, Department of Psychology, Kharazmi University, Tehran 1571914911, Iran

**Keywords:** psychological distress, virtual reality, COVID-19, well-being, digital therapeutics, self-help intervention

## Abstract

Background: The COVID-19 pandemic. In this framework, digital self-help interventions have the potential to provide flexible and scalable solutions for delivering evidence-based treatments that do not necessitate face-to-face meetings. Objective: as part of a multicentric project, the purpose of the current randomized controlled trial was to evaluate the efficacy of a Virtual-Reality-based self-help intervention (namely, COVID Feel Good) in lowering the psychological distress experienced during the COVID-19 pandemic in Iran. Methods: 60 participants were randomly assigned to the experimental (COVID Feel Good intervention group) or the control (no-treatment control group) condition. At the beginning of the intervention (Day 0), at the end of the intervention (Day 7), and after a 2-week follow-up (Day 21), measurements of depressive and anxiety levels, general distress, perceived levels of stress, hopelessness (primary outcome measures), perceived interpersonal closeness with the social world, and fear of COVID-19 (secondary outcome measure) were collected. The protocol consists of two integrated parts: the first part includes a relaxing 10-min three-hundred-sixty-degree (360°) video, while the second one includes social tasks with specified objectives. Results: In terms of the primary outcomes, participants in the COVID Feel Good intervention group improved in depression, stress, anxiety, and perceived stress but not hopelessness. Secondary outcome results showed an improvement in perceived social connectedness and a substantial decrease in fear of COVID-19. Conclusions: these findings on the efficacy of COVID Feel Good training add to the growing body of evidence demonstrating the feasibility of digital self-help interventions in promoting well-being during this unique period.

## 1. Introduction

The COVID-19 pandemic has substantially hindered healthcare delivery. On the one hand, well-known barriers to traditional face-to-face psychological treatments, such as the availability of qualified healthcare practitioners, expensive treatment costs, and stigma, have been compounded by social distancing policies adopted to contain the spread of the virus [1,2,3].

On the other hand, the pandemic is generating a significant need for treatment options able to support mental health and alleviate psychological discomfort [4,5]. Numerous longitudinal and cross-sectional studies revealed a considerable rise in psychological suffering during the first few months of the pandemic, which was most prominent among young individuals, females, and parents of children under the age of five [6]. Compared to minor changes in anxiety symptoms and general mental health functioning, increases in depressive symptoms were more pronounced and lasting [7]. These findings suggest a “mental health curve” is developing globally, as the prevalence of mental health conditions has increased considerably since the pandemic began [8,9,10].

In this framework, digital self-help interventions have the potential to provide flexible and scalable solutions for delivering evidence-based treatments that do not necessitate face-to-face meetings [11,12,13,14,15,16,17].

A consistent body of literature suggests that digital self-help interventions are effective in several clinical populations, including treating depressive symptoms [12,18,19] and anxiety disorders [20,21,22]. Some studies have already looked into the effectiveness of digital self-help interventions in reducing psychological distress during the pandemic. For instance, Wei et al. [23] tested the efficacy of a 15-day Internet-based self-help intervention for COVID-19 patients experiencing psychological distress. The intervention included different techniques, such as breathing exercises, mindfulness, and self-care techniques. They discovered that the intervention effectively reduced anxiety and depressive symptoms. Wahlund et al. [24] evaluated a 3-week Internet-based self-help intervention for pandemic-related dysfunctional anxiety in the general population. The training (based on cognitive-behavioral therapy) reduced pandemic-related dysfunctional anxiety and improved general mood and sleep disturbances. However, these studies involved small sample sizes, and more evidence should be collected about the effectiveness of such digital interventions in promoting well-being during this unique period.

In this scenario, among all the tools available for delivering efficient and engaging self-help training, Virtual Reality (VR) may have a significant position [25,26,27]. Beyond its traditional use as a “simulative instrument” [28] to immerse users in feared situations while progressively allowing the anxiety to lessen, VR could be exploited as a “safe place” [29]. Since the lockdown period eliminated “places” from our daily life, with serious consequences on the possibility to cultivate the “place attachment,” which is the cognitive–emotional relationship we build with a significant environment [28], the effectiveness of VR as a safe retreat to boost nature exposure during the COVID-19 pandemic has proven to be beneficial [30,31].

To this aim, our group designed the COVID Feel Good intervention [29], a self-help VR-based intervention. This training allows participants to immerse themselves in a naturalistic and beautiful “place”, allowing for virtual access to “places” that are no longer accessible and the dispersal of daily stressors encountered during the pandemic [32]. Here, they are taught specific relaxation techniques. The protocol, in particular, consists of two integrated parts: a 10-min three-hundred-sixty-degree (360°) video titled “The Secret Garden” is shown daily for one week, and this experience is supplemented with social tasks with specific objectives that are designed to be completed with another relevant person. We conducted two preliminary effectiveness studies in Italy [33] and Germany [34] and a multicentric European study [35] in four countries to investigate the efficacy of our self-help virtual therapeutic experience in lowering the psychological burden experienced during the pandemic lockdowns. These preliminary investigations demonstrated a constant reduction in perceived stress following participation in the COVID Feel Good intervention.

To further validate the efficacy of our intervention, we conducted a randomized controlled trial between April 2021 and September 2021 in Iran. On 19 February 2020, Iran, located in the Middle East area, announced the first verified case of COVID-19 from the city of Qom [36]. Iran was the most affected country in the world until 19 August 2020, with over 340,000 cases of COVID-19 and over 19,000 deaths [37]. Literature identified a severe mental health concern in the Persian population during the outbreak, highlighting the urgent need for effective and scalable mental health interventions [37]. For this trial, participants were randomly assigned to the experimental (COVID Feel Good intervention group) or control (no-treatment control group) conditions. At the beginning of the intervention (Day 0), at the end of the intervention (Day 7), and after a 2-week follow-up (Day 21), measurements of depressive and anxiety symptoms, general distress, perceived levels of stress, hopelessness (primary outcome measures), perceived interpersonal closeness with the social world, and fear of COVID-19 (secondary outcome measure) were collected (Day 21). We predicted that, when compared to responses from participants in the control group, the COVID Feel Good intervention would result in a reduction of depressive and anxiety levels, general distress, perceived levels of stress, and hopelessness (primary outcome measures), as well as an increase in perceived interpersonal closeness with the social world and a reduction of fear of COVID-19 (secondary outcome measures). At a 2-week follow-up, we anticipated that treatment gains would still be present.

## 2. Materials and Methods

### 2.1. Recruitment and Experimental Design

This was a parallel-group (ratio 1:1), randomized controlled study to investigate the efficacy of a novel self-help training program (namely, COVID Feel Good intervention) in reducing the psychological distress associated with the COVID-19 pandemic and related social distancing measures. Participants were recruited between April 2021 and September 2021 via advertisement. A total of 80 individuals were contacted for the screening. To be eligible, participants were required to meet the following inclusion criteria: (1) age at least 18 years or older; (2) to have sufficient knowledge of the Persian language; (3) to have experienced at least two months of social distancing measures related to the pandemic in Iran; (4) availability of a relevant partner for carrying out the social tasks; (5) availability of a smartphone with Internet access; (6) and to have normal or corrected-to-normal vision. Exclusion criteria (all self-reported) included a major a mental illness diagnosis, the absence of stereoscopic vision, and a balance/vestibular difficulty issue that would impair the VR experience. A total of 60 participants fulfilled all the above-mentioned inclusion/exclusion criteria and were randomized (ratio 1:1) to the COVID Feel Good intervention or no-treatment control group. Randomization was performed using computer-generated randomization via MS Excel.

Both groups completed all measures (see section Outcome Measures) on three occasions: at the beginning of the intervention (Day 0), at the end of the intervention (Day 7), and after a 2-week follow-up (Day 21). All the participants used an online platform to complete the assessment battery.

### 2.2. Ethics

This randomized controlled trial was conducted with the approval of the Review Board and the Ethics Committee of the Institute for Cognitive Science Studies (IR.UT.IRICSS.REC.1401.018). Trial registration: ISRCTN63887521.

### 2.3. Treatment Protocol

**Figure 1:**
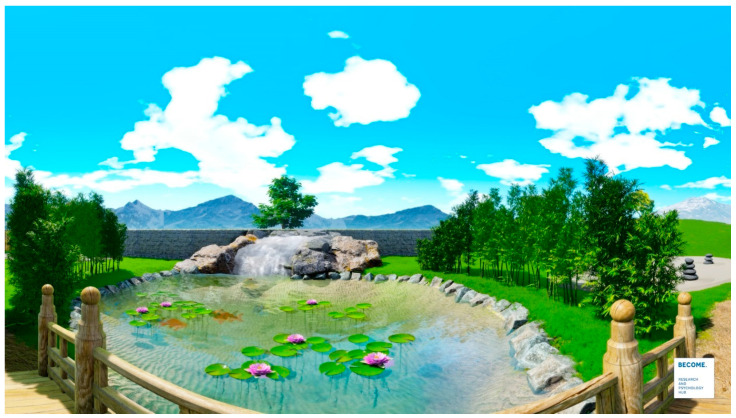
The “Secret Garden” is a 360° Virtual Reality scenario in which participants are immersed in a naturalistic and safe digital environment, away from the stressful conditions encountered in everyday life, where they can learn to relax and reflect on their experience through a guided procedure.

Participants in the intervention group received a one-week self-help training program called COVID Feel Good, which was designed to alleviate psychological distress caused by the COVID-19 pandemic and related social distancing measures [29]. COVID Feel Good is a daily intervention that includes seven thematic modules. Each module has two integrated parts. The first part of each module consisted of watching a 10 min 360° VR video titled “Secret Garden,” and the second part included seven different social tasks, with a different purpose for each day of the week. The virtual environment “The Secret Garden” (Figure 1) was created using the software Unreal Engine and can be experienced in both immersive (namely, using a head-mounted display or low-cost cardboard headset connected to a smartphone) and non-immersive modality (for example, both YouTube’s Android app and website accept 360° video formats). Participants had the opportunity to be immersed in a lovely and relaxing Japanese garden, with all the natural elements found in natural settings, such as the flow and the sound of running water. This experience was accompanied by a relaxation induction narrative, which was built according to compassion-focused therapy principles. The objective of this relaxation narrative was to activate the calming system while deactivating the human threat defense system, with a focus on providing and receiving care. The second part of each module included seven social tasks focusing on (a) emotion regulation skills, (b) strengthening resilience and coping skills, (c) assisting participants in self-monitoring and self-esteem protection, and (d) supporting participants in finding a personal meaning even in difficult times. For a detailed description of the modules, see Table 1. All the exercises were designed to be completed with another relevant person (though not necessarily physically together) to aid in the process of identifying and restructuring thought patterns as well as increasing social connectedness. These tasks took about 10 min to be completed; therefore, each module lasted approximately 20 min.

### 2.4. Outcome Measures

#### Primary Outcome Measure

Depression Anxiety Stress Scale (DASS-21): DASS-21 is a short version of the self-report instrument originally created and validated by Lovibond et al. [38] for evaluating depressive, anxiety, and stress feelings. It consists of 21 items, with 7 items assigned to each of the three subscales: depression (DASS-21 Depression), anxiety (DASS-21 Anxiety), and stress (DASS-21 Stress). Each item is scored on a scale from 0 (“did not apply to me at all”) to 3 (“applied to me very much”). Total scores for each of the three subscales are calculated. The DASS-21 has been translated and validated in Persian, with an acceptable test–retest reliability (r = 0.74–0.88) for all the three dimensions. Moreover, the Cronbach’s alpha coefficient was acceptable for anxiety (0.79), stress (0.91), and depression (0.93) [39].

Perceived Stress Scale (PSS) [40,41]: PSS is a self-report questionnaire used to evaluate people’s perceptions of stressful events. The scale is made up of 10 items on a 5-point Likert scale, and it assesses how stressful our daily experiences were perceived in the previous month. Participants in the current study were asked to rate their perceived level of stress in the previous two weeks. It produces a composite perceived stress score based on the sum of the different items and responses ranging from 0 to 40. The Cronbach’s alpha coefficient for the Persian questionnaire was acceptable (r = 0.90) [41].

Beck Hopelessness Scale (BHS): BHS [42] is a self-report questionnaire that evaluates pessimistic beliefs or a negative mood toward the future in three aspects of life: perceptions about the future, loss of drive, and general expectations. The Persian version [43] comprises 20 multiple-choice questions on a 5-point Likert scale (1—totally agree; 2—agree; 3—no idea; 4—disagree; 5—totally disagree), with higher scores indicating greater levels of hopelessness. The Persian version of BHS has good internal consistency (Cronbach’s alpha = 0.79).

### 2.5. Secondary Outcome Measures

Participants were also assessed at three time intervals with the following measures:

Social Connectedness Scale (SCS): SCS [44] is a self-report instrument that assesses an individual’s sense of connection to others or the social context. The questionnaire consists of eight items on a 6-point Likert scale. Composite scores can range between 0 and 48, with higher values suggesting a stronger feeling of social connectedness. The Cronbach’s alpha coefficient of the Persian version was acceptable (r = 0.87).

Fear of Coronavirus (FCOR) [45]: FCOR is a short self-report scale designed to assess fear experienced during the COVID-19 pandemic (“I am most afraid of coronavirus-19”). FCOR is composed of 7 items on a 5-point Likert scale. It produces a composite score and can range between 0 and 35, with higher values indicating greater fear of COVID-19. The Persian version of FCOR has high internal consistency (Cronbach’s alpha = 1.0).

### 2.6. Power

Sample size calculation was computed using the software G*Power (3.1) with a medium effect size (f = 0.25), a power of 0.95, and an alpha of 0.05. For a between-subject design, a minimum total sample size of 54 is suggested. However, given that we did not rule out the possibility that some participants dropped out during the intervention, we decided to recruit 60 participants.

### 2.7. Data Analysis

Before analysis, skewness and kurtosis coefficients of all variables were inspected for not-normal data distributions. Following that, a series of independent samples *t*-tests were run, which revealed no significant between-group baseline differences in outcome variables, indicating that randomization was successful. Then, to evaluate group changes (COVID Feel Good intervention vs. control group) across all three time points (Time—baseline assessment, T0; postintervention assessment, T1; two-week follow-up assessment, T2), we used the module GAMLj, which uses the R formulation of random effects as implemented by the function lme4, an R package, in Jamovi software. Consequently, we built separate linear mixed models for the PSS total score, DASS_21 subscales (DASS_21 Depression; DASS_21 Anxiety; DASS_21 Stress), BHS Total Score, SCS Total Score, and FCOR Total Score, using participants as random effects. Within-between subject changes were first evaluated by ANOVA F omnibus test employing the Satterthwaite approximation of degrees of freedom. Significant effects were examined with post hoc comparisons (Bonferroni’s adjustment) and are reported with estimated marginal means (EEM) and standard error (SE). All statistical analyses were carried out using Jamovi software.

## 3. Results

Table 2 and Table 3 show the demographic characteristics of the sample.

### 3.1. Primary Outcome Measures

Table 4 displays the means for primary and secondary outcomes across each assessment, divided into two groups (COVID Feel Good intervention vs. control group).

The COVID Feel Good group and the control group did not differ on any of the psychological questionnaire data at baseline (see Table 5; all *p*’s < 0.05).

**Figure 2:**
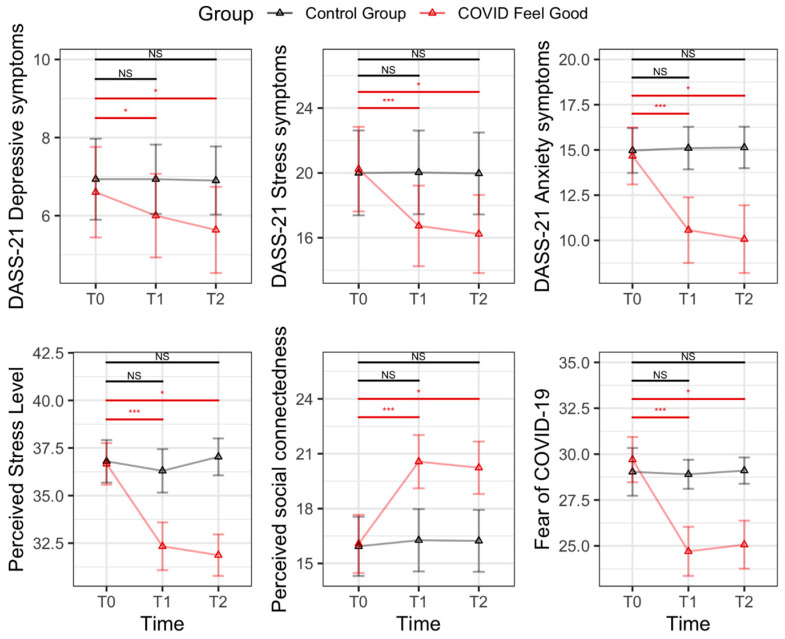
Pairwise comparisons for the DASS-21 Depression Subscale, DASS-21 Anxiety Subscale, DASS-21 Stress Subscale, Perceived Stress Scale (PSS), Social Connectedness Scale (SCS), and Fear of Coronavirus outcome (FCOR) across the different time intervals (T0—baseline; T1—end of the intervention; T2—2-week follow-up) divided by group (COVID Feel Good intervention vs. control group). NS: not significant; * *p* < 0.05; *** *p* < 0.001.

Analysis of variance (ANOVA) on the LMM’s parameters on the individual perceived level of stress revealed a significant main effect of time [F(2, 116) = 43.3, *p* < 0.001)] and group [F(1, 58) = 19.9, *p* < 0.001)]. Moreover, a significant interaction effect of time × group was found [F(2, 116) = 40.6, *p* < 0.001)] (see Figure 2). Bonferroni post hoc comparison showed a significant decrease in stress levels from T0 to T1 in the COVID Feel Good group (*p* < 0.001) but not in the control group. Moreover, the improvement observed in the intervention from T0 to T1 was maintained from postintervention to the 2-week follow-up (see Figure 2). Regarding DASS-21 subscales, first of all, results revealed a main effect of time for depression [F(2, 116) = 7.47, *p* < 0.001)] and stress symptoms [F(2, 116) = 52.20, *p* < 0.001)]. More importantly, a significant time x group interaction for both depressive [F(2, 116) = 6.59, *p* = 0.002)] and stress levels [F(2, 116) = 51.83, *p* <0.001)] emerged.

Post hoc comparison showed a significant decrease in depressive symptoms (*p* = 0.022) and stress levels (*p* < 0.001) from T0 to T1 in the COVID Feel Good group which were maintained at the follow-up assessment (*p* > 0.05). No changes were observed in the control group (all *p*’s > 0.05). Regarding the anxiety subscale of the DASS-21, results revealed a significant main effect of time F(2, 116) = 39.4, *p* < 0.001)] and group [F(1, 58) = 11.2, *p* < 0.001)] and, more importantly, an interaction effect time × group [F(2, 116) = 45.3, *p* < 0.001)]. Post hoc comparisons yielded significant findings, with decreases in anxiety levels from T0 to T1 for participants in the intervention group (*p* < 0.001). In addition, the improvement observed in the intervention from T0 and T1 was maintained from postintervention to the 2-week follow-up (see Figure 2). On the other hand, no differences in anxiety levels emerged across the different time points for the control group (*p* > 0.05). Regarding perceived hopelessness (as measured by the BHS), no significant differences emerged between the two groups and across the different time points (all *p*s > 0.05). Importantly, no interaction time x group was found [F(2, 116) = 0.315, *p* = 0.731)].

### 3.2. Secondary Outcome Measures

Analysis of variance (ANOVA) on the LMM’s parameters on the perceived level of social connectedness revealed a significant main effect of time [F(2, 116) = 24.64, *p* < 0.001)] and group [F(1, 58) = 7.83, *p* = 0.007)]. More interestingly, a significant interaction effect of time × group was found [F(2, 116) = 18.37, *p* < 0.001, see Figure 2). Bonferroni post hoc comparison revealed a significant improvement in social connectedness from T0 to T1 in the COVID Feel Good group (*p* < 0.001) but not in the control group. Moreover, the improvement observed in the intervention group was maintained from postintervention to the 2-week follow-up (see Figure 2). Finally, results revealed a significant interaction of time [F(2, 116) = 49.8, *p* < 0.001)] and group [F(1, 58) = 12.2, *p* < 0.001)] and an interaction effect of time × group [F(2, 116) = 48.0, *p* < 0.001)] for the fear of COVID-19. Post hoc comparisons indicated that participants in the intervention group experienced a decrease in their level of fear from T0 to T1 (*p* < 0.001), and this improvement was stable until the two-week follow-up assessment (see Figure 2). No improvements were observed among the different assessment points for participants in the control group.

## 4. Discussion

The objective of the current randomized controlled trial was to determine whether a novel Virtual Reality (VR) self-help training (namely, the COVID Feel Good intervention) protocol could help people in Iran cope with the psychological distress associated with the COVID-19 pandemic and related social restriction measures.

Consistent with previous findings [34,35], we found that the COVID Feel Good intervention was effective at reducing the psychological distress experienced during the COVID-19 pandemic in a Persian population sample. Our self-help training is based on the immersive 360° video “Secret Garden,” which allows participants to explore a lovely and natural setting while being guided by a validated technique to induce relaxation and self-reflection. Each day, participants were invited to visit the virtual garden, and then they were asked to complete seven social tasks (with specified objectives) with another significant partner to improve social connection with other individuals. In terms of primary outcome measures, participants in the COVID Feel Good intervention group improved in depression, stress, and anxiety symptoms and perceived stress but not in perceived hopelessness. Secondary outcome results showed an improvement in perceived social connectedness and a substantial decrease in fear of COVID-19. The results showed that the gains obtained with the participation in the COVID Feel Good intervention were maintained throughout the two weeks of follow-up. As observed before, these findings are consistent with our previous results demonstrating the effectiveness of COVID Feel Good in lowering psychological distress during the lockdown in a sample of Italian and German participants [33,34]. Similarly, in the Italian study we found no effect of the intervention on subjective feelings of hopelessness. According to Cipolletta and Ortu [46], the COVID-19 pandemic and associated restrictive measures have had enormous psychological effects, one of which is the suspension of time and our future [10]. This might have significantly worsened people’s feelings of hopelessness and pessimism.

These findings on the efficacy of COVID Feel Good training add to the expanding body of evidence showing the usefulness of digital therapeutics in addressing mental health symptoms and fostering well-being [15,20,47,48,49,50,51]. Because self-help interventions may be delivered through several media, including VR, they provide a possible easy-to-use and scalable solution to the COVID-19 mental health issue [52,53]. We have indeed used VR to offer participants the opportunity to explore a naturalistic and beautiful space [29], giving them virtual access to “places” that were inaccessible during the pandemic and instructing them in the acquisition of evidence-based relaxation methods. VR has already been used in self-help training for treating phobias and anxiety symptoms [20,54,55]. Still, the benefit of VR as a “safe retreat” to boost nature exposure was recently revealed in different studies during the COVID-19 pandemic [30,31,56]. Frost and colleagues [31], for example, examined the psychological effects of virtual nature immersion. They looked at 21 pieces of research with a total of 1301 participants and found that virtual immersion in nature considerably reduced negative affect.

As part of a larger multicentric project [35], the findings of this study provided more evidence for the protocol’s effectiveness across different contexts and countries. The proposed protocol has several advantages for adequately supporting individuals dealing with mental health issues related to the COVID-19 pandemic: it provides easy, self-guided training that can be accessed via a variety of digital platforms. In addition, the intervention is now available (https://www.covidfeelgood.com/) (accessed on 20 April 2022) in 16 different languages—English, Spanish, French, Brazilian/Portuguese, German, Italian, Turkish, Japanese, Korean, Farsi, Romanian, Catalan, Estonian, Polish, Russian, and Ukrainian. This gives participants around the world access to a free and adaptable tool for coping with the psychological burden brought on by the COVID-19 pandemic.

Since the literature suggests that a pandemic can have long-term psychological consequences such as anxiety, depression, and post-traumatic stress disorder in the general population [57], continued research and effort are needed to develop and test effective evidence-based strategies for enhancing mental health worldwide [21].

### Limitations

There are limitations to our study that must be noted. First, we did not include an active control condition. Therefore, despite positive effects being observed in the experimental group, definite conclusions about the efficacy of our intervention have yet to be reached. Second, because only short-term follow-up effects were studied, no conclusion about the long-term consequences of the intervention can be drawn. Longer follow-up studies are needed to determine how long treatment effects last. Third, to be consistent with our previous studies, we did not use any of the psychological measures targeted by the intervention as outcome measures, such as COVID-19 stress-related measures. Future research should also include COVID-19 stress-related responses as outcome measures to evaluate the efficacy of the intervention in reducing dysfunctional worry related to the pandemic or an abnormal stress-related response to the pandemic. Another critical aspect of future multicentric trials will be the investigation of potential cultural differences in how participants may experience VR. Finally, we did not control for how pandemic conditions may have affected outcomes during the three-week study period.

## 5. Conclusions

The logistical and economic challenges associated with traditional mental health care may have exacerbated COVID-19’s negative psychological consequences. There is an urgent need to design and adequately test digital self-help psychological interventions that are easily accessible without any constraints, thus providing first-aid psychological care to the general population. Overall, our results support the efficacy of our self-help VR-based intervention and add to the growing body of evidence supporting the use of digital therapeutics to alleviate psychological distress among the general population during the COVID-19 pandemic.

## Figures and Tables

**Table 1:** COVID Feel Good exercises.

Session	Exercise
Day 1: Fight rumination	Participants were asked to imagine themselves as a different person—a nurse who must care for a patient during his or her final moments of life, a doctor who must treat a patient, a politician who must decide—and write down their emotions and what they would do.
Day 2: Self-esteem improvement	Participants were asked to write down the five aspects of their personality that they are proud of and value.
Day 3: Encourage people to use episodic memory to create a consistent sense of self	Participants were asked to write four moments and/or events in their lives that have helped them become who they are, as well as a specific moment during the COVID-19 emergency.
Day 4: Increase in the sense of community	Participants were invited to name the five most important people in their lives.
Day 5: Encourage conscious self-regulation and self-organization of life objectives.	Participants were encouraged to write down three concrete goals and two dreams/aspirational goals that they hoped to achieve after the quarantine.
Day 6: Empathy empowerment	Participants were asked to consider the most recent major interaction they had with each of the five people they named on Day 4 and write down the emotions they felt at the time.
Day 7: Encourage a long-term psychological change.	Participants were asked to write down three parts of their lives with which they were dissatisfied and then, on a separate sheet, list the possible options in order of likelihood of success and cost/opportunity. On a separate sheet, they were asked to list probable issues and their consequences.

**Table 2:** Descriptive statistics of the participants who were randomly assigned to the COVID Feel Good intervention (N = 30).

	Mean	Std. Deviation
Age—Years	49.1	10.92
Gender		
Female	18	
Male	12	
Education (*N*)	Diploma (5), bachelor’s degree and above (25)	
Marital Status (*N*)	Single (2), Married (28)	

Note. Diploma = completed high school.

**Table 3:** Descriptive statistics of the participants who were randomly assigned to the no-treatment control group (N = 30).

	Mean	Std. Deviation
Age—Years	49.70	10.40
Gender		
Female	15	
Male	15	
Education (*N*)	Diploma (10), bachelor’s degree and above (20)	
Marital Status (*N*)	Single (3), Married (27)	

Note. Diploma = completed high school.

**Table 4:** Descriptive statistics for the outcome variables by group (COVID Feel Good intervention vs. control group) and time intervals (baseline, T0 Day 0, at the end of the intervention, T1 Day 7, and after a 2-week follow-up, T2 Day 21). Data are provided in means and standard deviation (SD).

		Primary Outcome Measures					Secondary Outcome Measures	
Group	TIME	Perceived Stress Level	Depressive Symptoms	Anxiety Symptoms	Stress Symptoms	Perceived Hopelessness	Social Connectedness	Fear COVID-19
COVID Feel Good Intervention	Baseline T0	36.7 (2.93)	6.6 (3.1)	14.7 (4.22)	20.2 (6.99)	70.2 (15.3)	16.1 (4.27)	29.7 (3.3)
	Post-intervention T1	32.3 (2.37)	6 (2.86)	10.6 (4.86)	16.7 (6.66)	67.7 (16.3)	20.6 (3.9)	24.7 (3.58)
	Two-week follow-up T2	31.9 (2.92)	5.63 (2.95)	10.1 (5.02)	16.2 (6.45)	67.3 (16.1)	20.2 (3.84)	25.1 (3.5)
Control Group	Baseline T0	36.8 (2.99)	6.93 (2.78)	15 (3.32)	20 (7.01)	70.8 (14.8)	15.9 (4.34)	29 (3.48)
	Post-intervention T1	36.3 (3.06)	6.93 (2.38)	15.1 (3.32)	20 (6.91)	70.7 (15)	16.3 (4.57)	28.9 (2.12)
	Two-week follow-up T2	37 (2.59)	6.9 (2.34)	15.1 (3.07)	20 (6.76)	71 (14.7)	16.2 (4.54)	29.1 (1.92)

**Table 5:** Baseline differences for the outcome variables between the two groups (COVID Feel Good intervention vs. control group).

	*t*	df	*p*
Primary Outcome Measures			
Perceived Stress Level	−0.175	58	0.862
Depressive Symptoms	−0.438	58	0.663
Anxiety Symptoms	−0.306	58	0.761
Stress Symptoms	0.129	58	0.898
Perceived Hopelessness	−0.163	58	0.871
Secondary Outcome Measures			
Social Connectedness	0.12	58	0.905
Fear COVID-10	0.761	58	0.45

## Data Availability

The datasets generated during and/or analysed during the current study are available from the corresponding author on reasonable request.
